# The adverse effect of overweight in assisted reproduction treatment outcomes

**DOI:** 10.5935/1518-0557.20170041

**Published:** 2017

**Authors:** Antonella Valeria Sampo, Celina Palena, Luciano Ganzer, Virginia Maccari, Gustavo Estofán, Mariana Hernández

**Affiliations:** 1 CIGOR - Centro Integral de Ginecología, Obstetricia y Reproducción. Córdoba, Argentina.

**Keywords:** body mass index, ICSI, ovarian stimulation, pregnancy rate

## Abstract

**Objective:**

To assess Body Mass Index (BMI) effects on the results obtained from ICSI cycles.

**Methods:**

We studied 266 ICSI cycles performed between January 2014 and December 2016. The patients were grouped according to their BMI in: Normal (18.5-24.9), Overweight (25.0-29.9) and Obese (>30). We compared the following variables between the groups: number of antral follicles, ovarian stimulation length, gonadotropin dose used, maximum estradiol level, follicles developed/antral follicles, retrieved oocytes/developed follicles and mature/retrieved oocytes, normal fertilization rate, embryo achieved/normal fertilized oocytes, clinical pregnancy and implantation rates. We used the Kruskal-Wallis and the Chi square tests. *p*<0.05 was considered significant.

**Results:**

Normal, Overweight and Obese patients presented comparable values for number of antral follicles (11.6±5.4, 12.5±5.5, 12.2±5.7), ovarian stimulation length (7.5±1.4, 7.6±1.1, 7.8±1.3) and gonadotropin dose used (2043±489, 1940±536, 2109±605). Obese patients had lower values of estradiol (1560±610, 1511±635, 1190±466; *p*=0.018), developed follicles (81%, 76%, 70%; *p*<0.0001), and retrieved oocytes (91%, 90%, 84%; *p*=0.0017); and not significantly lower values of mature oocytes (82%, 82%, 77%; *p*=0.26). The groups had comparable fertilization rates (72%, 73%, 69%) and embryo achieved rates (67%, 63%, 72%). The normal group had higher, but not significantly higher pregnancy and implantation rates (43%, 40%, 38%, *p*=0.53; and 33%, 26%, 23%; *p*=0.11), and significantly higher ongoing pregnancy rates (37%, 33%, 33%, *p*=0.042).

**Conclusion:**

Increased BMI patients had impaired ovarian response and lower pregnancy rates in ICSI cycles.

## INTRODUCTION

Infertility is a complex disorder, that includes medical, psychological and economical aspects, and has been recognized as a public health problem by the World Health Organization (WHO) (Boivin *et al*., 2007; Inhorn, 2003). Infertility affects one in seven couples and a significant proportion of these cases are believed to be directly or indirectly related to body weight: initiation and maintenance of reproductive functions are associated, among other factors, to an optimal body weight in women (Talmor & Dunphy, 2015).

While the prevalence of infertility has remained constant over the past 20 years, obesity has become an increasing global epidemic, with approximately 1.6 billion overweight adults and over 400 million with obesity (WHO, 2006). The proportion of overweight and obese women has increased from 30% in 1980 to 38% in 2013 (Ng *et al.*, 2014). Latin America and the Caribbean are not strangers to this trend: in the region, more than half of the adult population is overweight, and among those, 20% suffer from obesity (FAO, 2016).

Extreme body weights affect reproductive function through changes in the hypothalamic-pituitary-gonadal axis, causing periods of oligo-anovulation, as well as menstrual disorders (Brewer & Balen, 2010; Giviziez *et al.*, 2016; Grodstein *et al.*, 1994; Sathya *et al.*, 2010). When the fat mass is too low, the secretion of gonadotropins and, consequently, the reproductive capacity is reduced. When fat mass increases, adiposity increases the peripheral aromatization of androgens in estrogen; in addition, there would be a concomitant decrease in the hepatic synthesis of the sex hormone binding protein (SHBG), leading to an increase in the levels of circulating steroids and hypersecretion of luteinizing hormone (LH), with consequent altered endocrine environment, leading to reduced folliculogenesis (Gosman *et al.*, 2006). It has been shown that obese women are less likely to conceive within the first year of stopping contraception, compared with normal weight women, both in natural conception and assisted reproduction cycles (Brewer & Balen, 2010; Hartz *et al.*, 1984; Douchi *et al.*, 2002).

Most obese women are not sterile; however, obesity would have a negative impact on their fertility. It would exert its effect on conception and implantation through a cumulative deterioration of several processes, affecting ovulation, oocyte maturation, endometrial development, uterine receptivity and implantation (Brewer & Balen, 2010), increasing time to conception and abortion rates (Robker, 2008). There is also a higher rate of complications during pregnancy, such as hypertension, preeclampsia, gestational diabetes, postpartum hemorrhage, fetal macrosomia, and neonatal morbidity and mortality (Aly *et al.*, 2010; Aune *et al.*, 2014; Bhattacharya *et al.*, 2007).

The global increase of obesity in the population at reproductive age calls for reviews concerning its influence on natural and assisted reproduction (ASRM, 2015; Oliveira, 2016). There are few studies that analyzed the effects of obesity in patients who underwent assisted reproduction treatments, and their results are controversial (Koatz & de Souza, 2013).

Our objectives are to determine if altered BMI affects the pregnancy rates of patients performing assisted reproductive treatments (ICSI), and to identify whether BMI interferes with the different stages of an ICSI treatment.

## METHODS

### Design

Retrospective cohort study.

### Population

We studied 274 patients who underwent ICSI cycles between January 2014 and December 2016.

The patients fulfilled the following inclusion criteria: age from 30 to 38 years, baseline FSH up to 10IU/L, 3 or more mature oocytes recovered and 1 or more viable embryos obtained, as well as having been transferred with 1 or 2 embryos.

The patients were excluded when they presented polycystic ovarian syndrome, organic uterine pathology (polyps, large intramural and submucous myomas, uterine malformations: unicornuate uterus, septate uterus, bicornuate uterus), grade IV endometriosis, or those whose partner had azoospermia or used a heterologous semen sample.

### Ovarian stimulation

Ovarian stimulation was performed under a GnRH antagonist and gonadotropins flexible protocol. Periodic ultrasound and serum estradiol controls were performed. The criterion for antagonist use was to have follicles ≥14 mm. When follicular development of three follicles of 18 mm or more was reached, 10,000IU of HCG was administered. Follicular aspiration was performed after 35-36 hours of HCG administration.

### Laboratory procedure

The culture of oocytes and embryos was performed in individual microdrops of sequential media under oil (Vitrolife). Semen samples were processed by Swim-up or Isolate gradients. ICSI was performed after 5-6 hours of aspiration. Oocyte survival and fertilization were assessed after 17 hours. Embryo quality and cleavage were analyzed at 41 and 65 hours. Good-quality embryos were those with symmetric or slightly asymmetric blastomeres, non-multinucleated at day 2, less than 20% fragments, expected number of blastomeres (4 at day 2, 6 or more at day 3). One or two embryos were transferred (the one showing best quality at the time of transfer) on day 2 or 3.

Biochemical pregnancy was determined by the presence of two positive and increasing doses of human chorionic gonadotropin beta subunit (ß-HCG) 14 days after oocyte recovery, and clinical pregnancy was determined by transvaginal ultrasound at four weeks after the transfer (equivalent to six weeks of pregnancy).

Height and weight were measured by the same examiner during the pre-surgical evaluation, performed together with the stimulation cycle. The height was measured to an accuracy of 0.5cm and the weight was measured with an accuracy of 0.1kg.

### Statistical analysis

Stratification of study groups according to BMI: the body mass index was calculated as the weight in kilograms divided by the square of the height in meters (kg/m^2^).

The population was divided into four groups according to the WHO's International BMI classification: Underweight (<18,5 kg/m^2^), Normal (18.5-24.9 kg/m^2^), Overweight (25.0-29.9 kg/m^2^) and Obese (≥ 30 kg/m^2^) (WHO, 2004).

The groups were compared taking in account:

1. Baseline characteristics of the patients: mean age, baseline FSH and LH and antral follicles count
2. Characteristics of ovarian stimulation: average of the required days of stimulation and the amount of gonadotropins used.
3. Ovarian stimulation results: the average of the maximum estradiol value, and the percentages of developed follicles/antral follicles, recovered oocytes/developed follicles, and mature oocytes/recovered oocytes.
4. Quality of oocytes obtained: estimated through total and normal fertilization rates after ICSI (total and normal fertilized oocytes/injected oocytes), percentage of total and good quality embryos obtained (total and good quality embryos obtained on day 3/normally fertilized oocytes),
5. Results obtained after embryo transfer: clinical pregnancy rates (clinical pregnancy/transferred patients), implantation rates (gestational sacs/embryos transferred), spontaneous abortion rate (abortions/clinical pregnancies), ongoing pregnancy rates (Ongoing clinical pregnancies/transferred patients).

Statistical analysis was performed using the Medcalc 10.2.0.0 software. The Kruskal-Wallis test was used to compare the averages between the groups and the chi-square test to compare the results expressed as percentages between the groups. A value of *p*<0.05 was considered statistically significant.

## RESULTS

Among the 274 patients included, 8 were underweight (3%), 190 were normal (69%), 52 were overweight (19%) and 24 were obese (9%). Of the 24 obese patients, 17 (75%) had type I obesity (BMI 30 to 34.99); 4 (21%) had type II obesity (BMI 35 to 39.99), and only 1 (4%) had type III obesity (BMI 40 to 44.99). The Underweight group of patients was excluded from the remaining analysis due to the low number of cases.

### Basal characteristics

The groups Normal, Overweight and Obese were comparable in terms of baseline characteristics: patient age, baseline FSH and LH, and antral follicle count (Table 1).

**Table 1:** Patients' characteristics according to their BMI

	Normal	Overweight	Obese	*P*
Patients	190	52	24	
BMI	21.8±1.6	27.1±1.5	32.8±2.8	<0.0001
Patients age	34.5±2.3	34.4±2.4	34.3±2.2	0.88
Baseline FSH	6.9±2.1	6.7±1.7	6.8±2.1	0.66
Baseline LH	5.3±2.3	5.3±1.7	4.5±1.6	0.34
Antral follicles number	11.6±5.4	12.5±5.5	12.2±5.7	0.50

Kruskal-Wallis test, statistically significant if *P*<0.05.

### Characteristics and results of ovarian stimulation

No difference was found in ovarian stimulation of the patients according to the BMI group, being comparable in days of stimulation required and quantity of gonadotropins used.

In contrast, the results of the stimulation were significantly different. The maximum value of estradiol decreased significantly with BMI.

Also, when analyzing the ovarian response obtained in relation to the antral follicle number of the patients, in the Normal, Overweight and Obese groups, we observed a statistically significant decrease in the percentage of developed follicles/antral follicles (1774/2190, 81%, 494/649, 76%; 205/293, 70%; *p*<0.0001), and in the percentage of recovered oocytes/follicles developed (1619/1774, 91%, 444/494, 90%, 172/205, 84%, *p*=0.0017).

The percentage of mature oocytes/recovered oocytes decreased in the obese group but this difference was not statistically significant (1333/1619, 82%; 365/444, 82%; 133/172, 77%; NS, *p*=0.26) (Table 2).

**Table 2:** Controlled ovarian stimulation, proportion of developed follicles and oocytes recovered according to patients' BMI

	Normal	Overweight	Obese	*P*
Antral follicles number	11.6±5.4	12.5±5.5	12.2±5.7	0.50
Days of ovarian stimulation	7.5±1.4	7.6±1.1	7.8±1.3	0.56
Gonadotropin dose used (IU)	2043±489	1940±536	2109±605	0.66
Maximum estradiol value (pg/ml)	1560±610	1511±635	1190±466	0.02
Developed/antral follicles	81%	76%	70%	<0.0001
Recovered oocytes/developed follicles	91%	90%	84%	0.0017
mature/recovered oocytes	82%	82%	77%	0.26

Data expressed as mean ± standard deviation, or as percentages. Kruskal-Wallis test or Chi-square test - statistically significant if *P*<0.05.

### Quality of oocytes obtained

We found no differences between the Normal, Overweight and Obese groups, in total fertilization rates (978/1245, 73%, 272/342, 74%, 103/127, 77%, *p*=0.64); normal fertilization rates (898/1245, 72%, 251/342, 73%, 89/127, 69%, *p*=0.74), total embryos obtained/normally fertilized oocytes (606/898, 67%, 158/251, 63%, 64/ 89, 72%, *p*=0.93) and good quality embryos obtained/ normally fertilized oocytes (565/898, 63%, 141/251, 56%, 61/89, 69%, *p*=0.07) (Table 3).

**Table 3:** Fertilization rate and embryonic development according to patients' BMI

	Normal	Overweight	Obese	*P*
Injected oocytes	1245	342	129	
Total fertilization rate	73%	74%	77%	0.64
Normal fertilization rate	72%	73%	69%	0.74
total embryos obtained	67%	63%	72%	0.93
good quality embryos obtained	63%	56%	69%	0.07

Chi-square test - statistically significant if *P*<0.05.

### Results obtained after embryo transfer

The quantity and quality of transferred embryos were comparable between the groups.

Clinical and ongoing pregnancy rates and embryo implantation rates showed a decrease as BMI increases, being statistically significant only for ongoing pregnancy (Table 4).

**Table 4:** Results obtained after the transfer according to patient BMI

	Normal	Overweight	Obese	*P*
Patients transferred	190	52	24	
Number of embryos transferred	318	91	40	
Mean of embryos transferred	1.7±0.5	1.8±0.4	1.7±0.6	0.55
Good quality embryos transferred	1.4±0.6	1.5±0.6	1.3±0.6	0.62
Clinical pregnancy rates	43%	40%	38%	0.53
Abortion rates	13%	19%	11%	0.77
Ongoing pregnancy rates	37%	33%	33%	0.042
Implantation rate	33%	26%	23%	0.11

Kruskal-Wallis test or Chi-square test, statistically significant if *P*<0.05.

## DISCUSSION

The results obtained in different retrospective studies and case series about the relationship between BMI and pregnancy achievement in patients undergoing assisted reproductive treatments (ICSI) have been contradictory. The number of patients included, the age of the patients and their levels of obesity operate as confounding factors when results are analyzed (Koatz & de Souza, 2013).

In our study, we obtained a homogeneous sample, excluding those patients who had polycystic ovarian syndrome, to avoid this confounding variable. A narrow age range, from 30 to 38 years, was used to avoid the confusion caused by the age dispersion (Koatz & de Souza, 2013). Patients with low weight were also excluded from our analysis, because we had few of them. The resulting groups were comparable in age, baseline FSH and LH values and number of antral follicles.

The obesity degree of our population is low, when compared with other studies. The 28% of our study population exceeded normal weight for their height, being 9% obese. Only 4% of obese were type III. In comparison, the 2008-2010 SART registry has an incidence of 53% of patients with overweight, being obese a 23% of these, 14% of the obese patients have type III or more obesity (Provost *et al.*, 2016b).

Ovarian stimulation had similar characteristics between the groups, with comparable values of days of stimulation and doses of gonadotropins used. Despite this, we noticed a statistically significant decreasing linear trend in total follicles at the end of the stimulation, recovered oocytes and mature oocytes obtained between the different groups, being higher in the Normal group and lower in Obese patients.

Our results coincide with studies carried out by other authors such as Matalliotakis *et al.* (2008) and Sarais *et al.* (2016), who also found a smaller number of antral follicles and lower oocyte recovery. On the other hand, other authors (Ozekinci *et al.*, 2015; Sathya *et al.*, 2010; Awartani *et al.*, 2009; Vilarino *et al.*, 2010) found no significant differences between these results when comparing the groups. Probably this may be because they did not exclude patients with polycystic ovarian syndrome from the population to be analyzed, which allows to obtain more oocytes from these patients, but of lower quality (Harris *et al.*, 2010; Ludwig *et al.*, 1999). Koatz & de Souza (2013), in their review, concluded that obese patients require higher doses of gonadotropins for comparable or lower responses, and that the required dose increases with the type of obesity. Since our obese population is mostly type I and II, it is possible that the requirement of gonadotropins is not significantly higher, although we did find lower responses and lower oocyte recovery.

In our population, despite recovering less oocytes in overweight and obese patients, the maturity and quality of the oocytes did not appear to be affected, with the groups presenting a similar percentage of fertilization, as well as embryo quantity and quality.

Some authors showed results comparable to ours at this stage of treatment (Koatz & de Souza, 2013; Ozekinci *et al.*, 2015; Sarais *et al.*, 2016; Sathya *et al.*, 2010; Vilarino *et al.*, 2010). Other authors (such as Awartani *et al.*, 2009; Matalliotakis *et al.*, 2008) report a smaller number of embryos obtained from obese patients. According to their results, this may be due to a lower initial number of oocytes than to a decrease in embryo development due to oocyte quality.

All patients in our study were transferred in the stimulation cycle. Embryo quantity and quality were comparable between the groups. We observed a decreasing outcome in pregnancy rates as BMI increases, where normal-weight patients had higher rates of clinical pregnancy and implantation (non-significant), and higher rates of ongoing pregnancy (statistically significant). Regarding the spontaneous abortion rate, we did not find significant differences between the groups, although the number of pregnancies was low to draw conclusions.

The results concerning pregnancy and abortion rates are the most controversial in the literature. The degree of obesity of the study population and the number of patients studied, among other factors, modify the results obtained. Some authors did not find significant differences in pregnancy rates (Awartani *et al.*, 2009; Matalliotakis *et al.*, 2008; Ozekinci *et al.*, 2015; Sathya *et al.*, 2010; Vilarino *et al.*, 2010); Koatz & de Souza (2013) showed in their studies, that five of them coincide in a decrease in pregnancy rates, whereas eight other studies did not report it.

Wang *et al.* (2002) found a higher rate of miscarriages in the obese patient group, and argued that the endocrinological and/or biochemical environment associated with obesity, such as insulin resistance, would bring about a hostile environment for oocytes and embryos at intraovarian and intrauterine levels.

The 2008-2010 SART registries, in more than 200,000 cycles with oocytes of their own, showed a decrease in pregnancy rates and implantation of around 1% for every 5 points in BMI (Provost *et al.*, 2016b). In egg donation patients, pregnancy rates according to the recipient's BMI show comparable values, and only a decrease is seen when BMI >40 in the recipient (Provost *et al.*, 2016a). This decrease would indicate the presence of intrauterine factors affecting the results, although it does so in patients with a BMI greater than that of our population.

## CONCLUSION

Our study indicates that, in patients with comparable antral follicle counts, those with overweight and obesity had a stimulation similar to that of normal weight patients (in terms of gonadotropin doses and days of stimulation) but achieved a significantly lower ovarian response (in the amount of developed follicles and recovered oocytes).

Despite this, there were no statistically significant differences between the three groups insofar as maturity is concerned, neither in oocytes recovered, nor in the rates of fertilization, and embryo quantity and quality.

Clinical pregnancy rates, ongoing pregnancy and implantation showed a decreasing trend when BMI increases. For ongoing pregnancies, this difference was statistically significant.

We concluded that the increase in BMI would have an adverse effect on reproductive outcomes in patients performing ICSI treatments. It is necessary to advise healthy habits, balanced diet and weight loss in overweight and obese women who desire pregnancy.
